# Spatial co-transcriptomics reveals discrete stages of the arbuscular mycorrhizal symbiosis

**DOI:** 10.1038/s41477-024-01666-3

**Published:** 2024-04-08

**Authors:** Karen Serrano, Margaret Bezrutczyk, Danielle Goudeau, Thai Dao, Ronan O’Malley, Rex R. Malmstrom, Axel Visel, Henrik V. Scheller, Benjamin Cole

**Affiliations:** 1 Joint Bioenergy Institute, Emeryville, CA USA; 2 Department of Plant and Microbial Biology, University of California Berkeley, Berkeley, CA USA; 3 Environmental Genomics and Systems Biology Division, Lawrence Berkeley National Laboratory, Berkeley, CA USA; 4 Joint Genome Institute, Lawrence Berkeley National Laboratory, Berkeley, CA USA; 5 School of Natural Sciences, University of California Merced, Merced, CA USA

**Keywords:** Arbuscular mycorrhiza, Transcriptomics, RNA sequencing

## Abstract

The symbiotic interaction of plants with arbuscular mycorrhizal (AM) fungi is ancient and widespread. Plants provide AM fungi with carbon in exchange for nutrients and water, making this interaction a prime target for crop improvement. However, plant–fungal interactions are restricted to a small subset of root cells, precluding the application of most conventional functional genomic techniques to study the molecular bases of these interactions. Here we used single-nucleus and spatial RNA sequencing to explore both *Medicago truncatula* and *Rhizophagus irregularis* transcriptomes in AM symbiosis at cellular and spatial resolution. Integrated, spatially registered single-cell maps revealed infected and uninfected plant root cell types. We observed that cortex cells exhibit distinct transcriptome profiles during different stages of colonization by AM fungi, indicating dynamic interplay between both organisms during establishment of the cellular interface enabling successful symbiosis. Our study provides insight into a symbiotic relationship of major agricultural and environmental importance and demonstrates a paradigm combining single-cell and spatial transcriptomics for the analysis of complex organismal interactions.

## Abstract (web summary)

Using a combination of single-cell and spatial transcriptomic analysis, Serrano et al. uncover transcriptional dynamics during the establishment of arbuscular mycorrhizal symbiosis.

## Main

Arbuscular mycorrhizal (AM) fungi occur in all major terrestrial ecosystems^1^. They are fundamental to agricultural production as they provide plants with nutrients, particularly non-renewable phosphorus, as well as resistance to abiotic stress^2^ and pathogens^3^. Plants reward these services by transferring carbohydrates and lipids to AM fungi, which enables extension of extraradical mycelium in the soil^4^.

Intense coordination between species is required for symbiotic recruitment, development and maintenance within the root. Preceding contact, signalling between plant roots and germinating fungal spores initiates hyphal branching towards the root at which a hyphopodium will form to initiate the symbiosis^5^. Upon contact, a plant-derived pre-penetration apparatus guides the fungus across and through the epidermis, with hyphae travelling both inter- and intra-cellularly to reach the inner-most cortical cells^6,7^. Hyphae then differentiate to form branched structures termed arbuscules within cortical cells^8^. The host plant restructures the cortex cell and builds a peri-arbuscular membrane (PAM)^4^, creating an apoplastic space across which metabolite exchange occurs between species. As symbiosis develops, the carbon supply from the root allows for expansion of an intraradical and extraradical mycelium, through which soil minerals are transported into the plant^5^.

Decades of research resulted in much progress regarding functional characterization of genes involved in the AM symbiosis^9–17^, yet many remain to be characterized. Multiple characteristics of AM symbiosis complicate traditional transcriptomic approaches. As obligate biotrophs, AM fungi cannot complete their life cycles or be cultured independently in asymbiotic conditions. Recent advances in asymbiotic sporulation of mycorrhizal fungi using bacterial fatty acids as stimuli are encouraging^18,19^, but it remains challenging to develop axenic AM inoculums. Due to asynchronous colonization, many developmental stages exist simultaneously within the cortex. This limits the ability of whole-root transcriptomics to differentiate between transcriptional profiles of each stage^20^. Arbuscules are extremely transient structures, lasting only a few days before senescence^21,22^, confounding efforts to distinguish discrete phases of interaction. Furthermore, arbuscule collapse and vesicle or spore formation indicates that the plant and fungus are assimilating exchanged nutrients, which creates the need to analyse root cells that appear to be non-colonized^23^. Several groups have elegantly addressed these challenges using laser-capture microdissection (LCM) to obtain transcriptomes of cortex cells visually confirmed to be directly adjacent to fungal appressoria (early stage) and colonized cortex cells (CCCs; late stage)^24,25^. One disadvantage of LCM is that it limits investigation to cell types already known to be involved, which creates the need for an unbiased approach to analyse all root cell types.

The rapid adoption of single-cell RNA sequencing (scRNA-seq) or single-nuclei RNA sequencing (snRNA-seq), with potential to identify novel cell types, model developmental trajectories and analyse transcriptional activity of individual cells^26^, has revolutionized plant biology. In both scRNA-seq and snRNA-seq, investigation of transcriptomes from all cell types is possible, rather than requiring manual selection of individual cells, as with LCM. Moreover, snRNA-seq’s rapid protocols are robust to diverse organisms and tissue types. In addition, certain cell types are preferentially released as protoplasts during enzymatic digestion for scRNA-seq, but nuclei are extracted uniformly across cell types, leading to a more representative population of cell types in snRNA-seq datasets^27,28^. For example, arbusculated cells have highly ramified cell membranes, which may be difficult to recover after enzymatic digestion due to their increased surface area. In both scRNA-seq and snRNA-seq, the spatial context of gene expression is lost upon dissociation of cells from the tissue. Spatial transcriptomics allows for sequencing of cell transcriptomes within the tissue context, adding a novel dimension to the data^29^.

In this Resource, we applied single-nuclei and spatial transcriptomics to the interaction between the model legume *Medicago truncatula* and the AM fungus *Rhizophagus irregularis* to create a two-dimensional integrated map of plant and fungal transcriptomes during symbiosis. We provide an unbiased spatial and single-nuclei transcriptomics dataset that profiled a multi-kingdom interaction. The spatially resolved transcriptome provides insight into coordinated gene expression occurring between the two partners across all major *M. truncatula* root cell types. This transcriptomic map represents a novel resource for AM fungi research and demonstrates the value of novel multi-omics approaches in answering biological questions.

## Results

### Nuclear RNA profiling identifies *M. truncatula* cell types

**Fig. 1:**
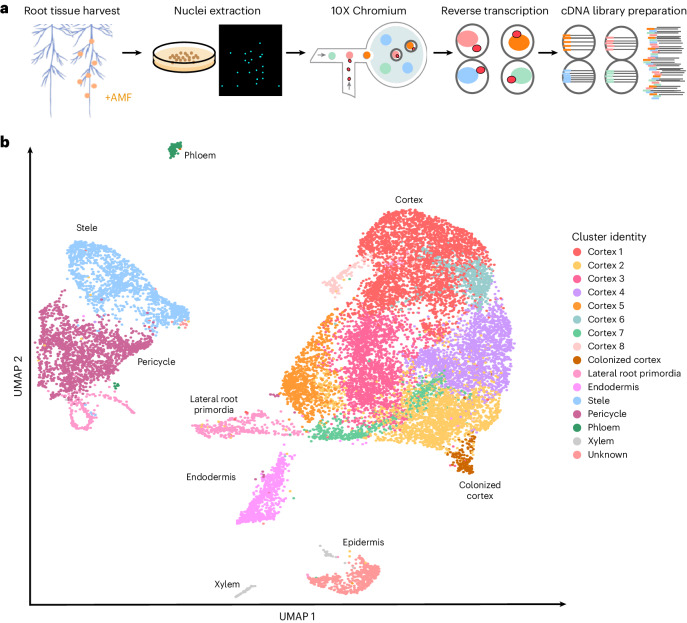
snRNA profiling in *M. truncatula* roots colonized by *R. irregularis.* **a**, An overview of the approach. *M. truncatula* root tissue is flash frozen for nuclei extraction and subsequent snRNA-seq using the 10x Genomics Chromium platform. Intact single nuclei are emulsified with gel beads containing barcoded oligonucleotides within a microfluidic chamber, resulting in a barcoded cDNA library after reverse transcription. **b**, The UMAP coordinates of 16,890 *M. truncatula* nuclei from three AM-colonized root harvest timepoints clustered by similarity in transcriptional profiles. The identities of 16 unique clusters are represented by different colours.

To gain a comprehensive transcriptional profile of the plant/AM fungal interaction, we performed snRNA-seq and spatial RNA-seq on *M. truncatula* roots, which were mock-inoculated or inoculated with the AM fungus *R. irregularis*. We isolated and purified nuclei from *M. truncatula* roots by fluorescence-activated nuclei sorting (FANS, Fig. 1a) before loading the suspension onto a microfluidic chip for snRNA-seq profiling^30^.

**Extended Data Fig. 1:**
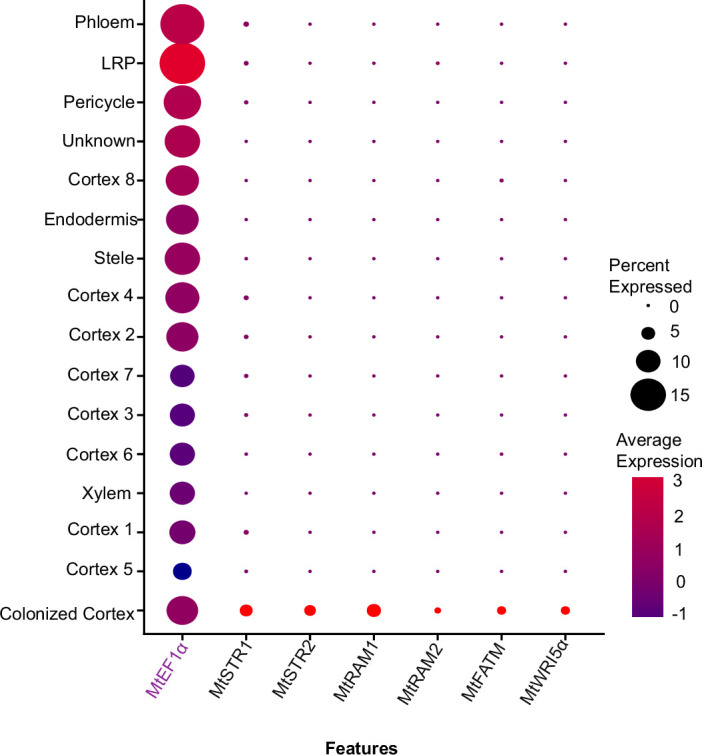
Select marker gene expression for single-nuclei clusters. Dot-plot of expression profiles for select *M. truncatula* marker genes for unique root cell types across labeled cluster identities utilizing hierarchical clustering within the integrated single-nuclei datasets.

Quality filtering and unsupervised clustering resulted in a dataset of 16,890 nuclei grouped in 16 distinct cell clusters (Methods). We assigned cluster identities using cell type-specific gene expression profiles derived from *A. thaliana* root single-cell datasets^31–35^ as well as a rhizobia-colonized *M. truncatula* single-nuclei dataset^36^ (Supplementary Table 1 and Extended Data Fig. 1). Nine clusters (11,298 cells) exhibited characteristics of cortex cell identity, with one additional cluster (174 cells) composed of cortex cells colonized by *R. irregularis* (Fig. 1b). We also identified all other major *M. truncatula* root cell types and generated marker gene sets for each (Supplementary Table 1).

### Simultaneous spatial capture of plant and fungal transcripts

**Fig. 2:**
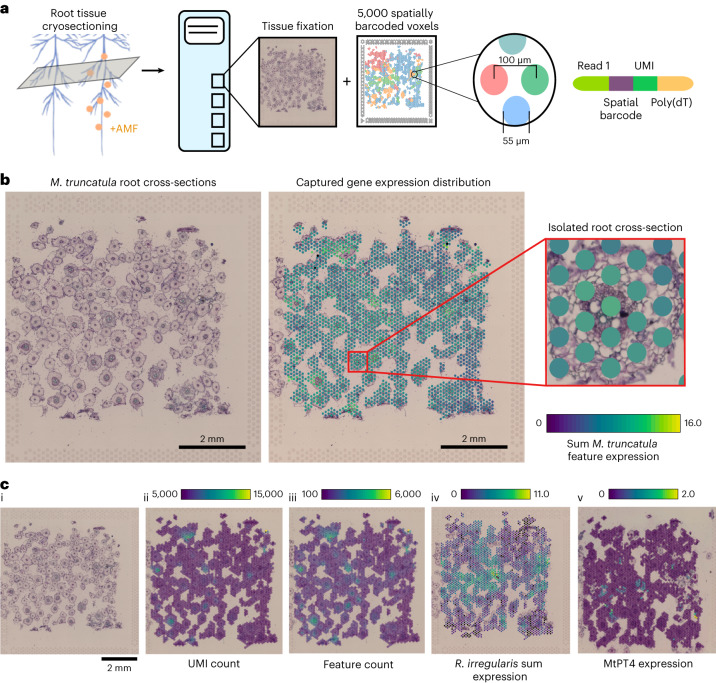
Spatial transcriptomics enables simultaneous capture of *M. truncatula* and *R. irregularis* transcripts*.* **a,** *M. truncatula* root tissue is flash frozen to create 16 µm thick cryosections, each containing numerous root cross-sections. Cryosections are fixed to capture areas, each of which is equipped with ~5,000 spatially barcoded voxels at a resolution of 55 µm. **b**, Side-by-side images of brightfield tissue image and underlying spatial capture voxels, with a close-up view of a single root cross-section within the capture area (one representative capture area out of nine mycorrhizal capture areas was analysed) highlighting voxel size in relation to the tissue. **c**, Capture area containing cross-sections from *M. truncatula* roots infected with *R. irregularis* at 28 dpi (one representative capture area out of nine mycorrhizal capture areas analysed). Image of root cross-sections within capture area (i). UMI count (ii) and feature count (iii) overlaid onto spots underlying tissue. Expression pattern of all *R. irregularis* transcripts captured (scale, log_2_ of UMI counts) (iv). Expression pattern of the arbuscule marker gene post-imputation (v), *MtPT4*, exhibiting overlap in spots with the highest expression of fungal transcripts (scale, log_2_ of UMI counts). Visualization done in Loupe Browser.

**Fig. 3:**
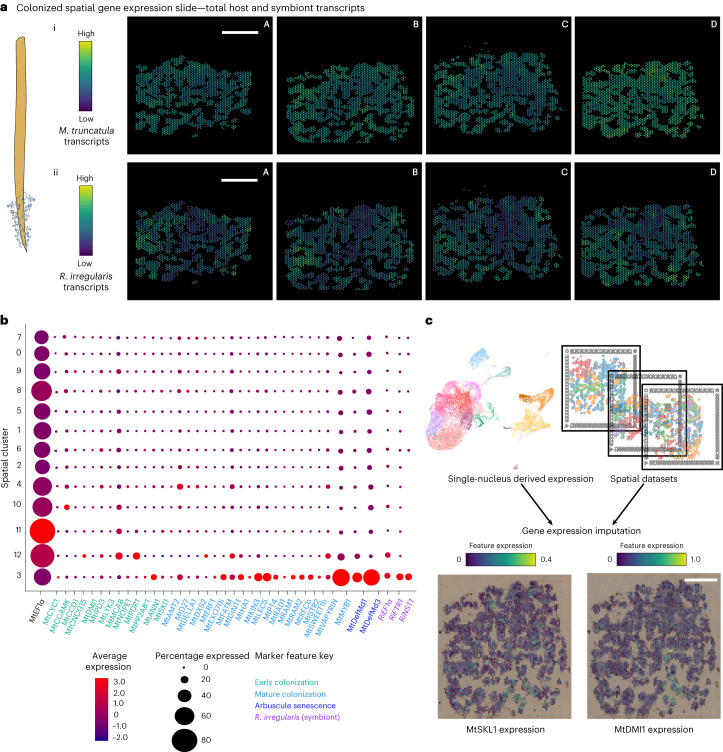
Transcriptomic profiling reveals coordinated gene expression between the symbiotic partners. **a**, The spatial distributions of host and symbiont transcript expression from four unique capture areas containing lateral root cross-sections from *M. truncatula* plants at 28 dpi (scale bar, 1 mm; scale, log_2_ of UMI counts). i, Four unique inoculated capture areas exhibiting the spatial distribution of all *M. truncatula* transcripts within the roots. ii, The same four capture areas exhibiting the spatial distribution of all *R. irregularis* transcripts within the roots. **b**, A dot plot of *M. truncatula* and *R. irregularis* housekeeping genes along with genes known to be involved in different stages of the symbiosis utilizing hierarchical clustering within the integrated mycorrhizal spatial object. **c**, A visualization of gene expression imputation from snRNA-seq mycorrhizal-integrated dataset for two lowly expressed genes, *MtSKL1* and *MtDMI*, within a single representative Visium Spatial Gene Expression capture area out of nine mycorrhizal-treated capture areas analysed (scale bar, 1 mm).

To investigate gene expression from both symbiotic partners, we performed spatial transcriptomic profiling on inoculated and mock-inoculated *M. truncatula* roots at 28 dpi (Fig. 2a,b and Supplementary Table 2). An example capture area from an inoculated plant displays numerous root cross-sections fixed and stained on the glass surface (Fig. 2c, i). On average, inoculated capture areas resulted in 20,333 and 5,084 transcripts mapping to the *M. truncatula*^37^ and *R. irregularis*^38^ genomes, respectively, while mock-inoculated capture areas resulted in an average of 21,987 (*M. truncatula*) and 23 (*R. irregularis*) transcripts. Spatial unique molecular identifier (UMI; Fig. 2c, ii) and feature (Fig. 2c, iii) distributions indicate a uniform capture of transcripts across the expected areas of each cryosection, with few hotspots of fungal colonization associated with increased transcript counts from *R. irregularis* (Fig. 2c, iv) and increased expression of phosphate transporter 4 (*MtPT4*), a marker gene for arbusculated cells^39^ (Fig. 1d, v). The spatial technology relies on polyadenylated transcript capture via oligo(dT) primer sequences, which enabled unbiased capture of *R. irregularis* and *M. truncatula* transcripts simultaneously. Analysing the distribution of all *M. truncatula* transcripts within each inoculated capture area (Fig. 3a, i) and comparing it with that of all *R. irregularis* transcripts (Fig. 3a, ii) showed distinct patterns of messenger RNA capture and expression between the two species, indicating the successful spatially resolved capture of transcriptomes during symbiosis.

### Overlapping, symbiosis-responsive transcriptomes

**Extended Data Fig. 2:**
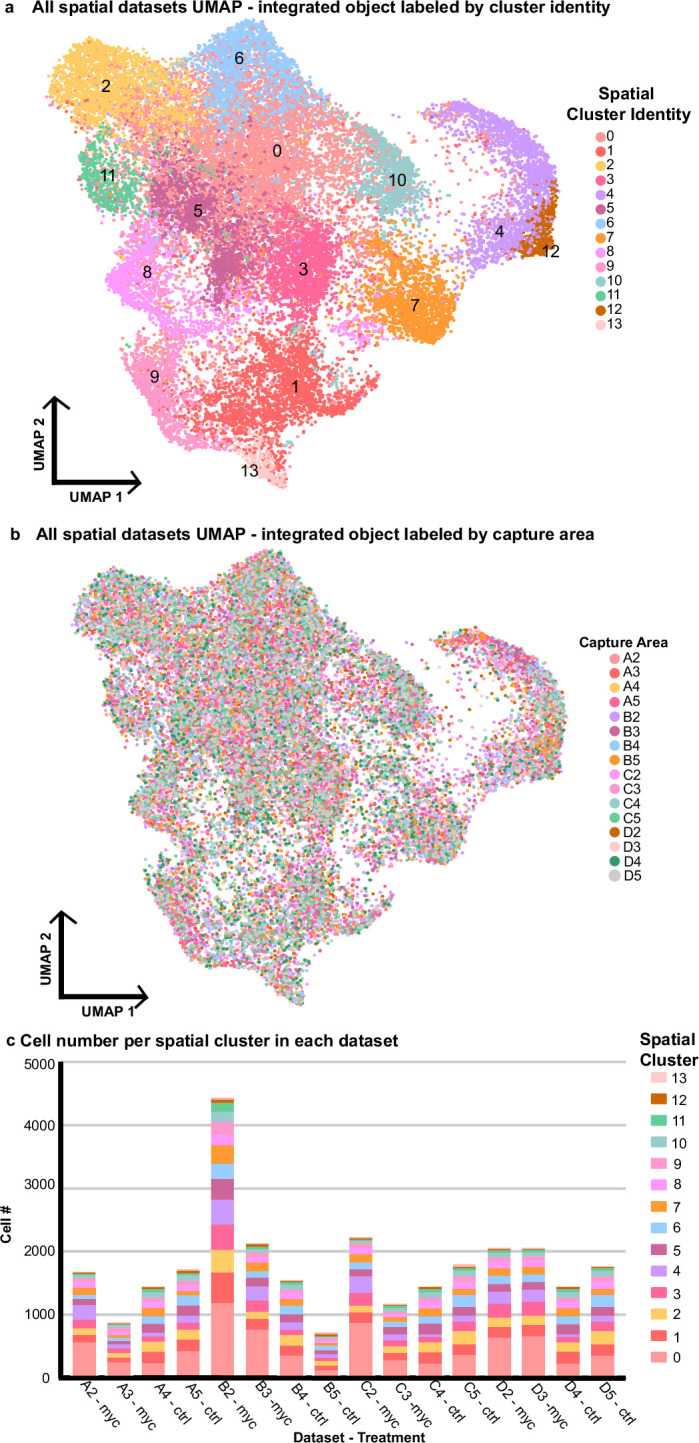
Spatial RNA-seq integrated UMAPs by treatment and dataset. **a**, UMAP of 28,564 spatial voxels from the integrated Seurat object of all root harvests. The identities of 17 unique clusters are represented by different colors according to cluster identity. **b**, UMAP of 28,564 spatial voxels from the integrated Seurat object of all root harvests. Different colors correspond to the original dataset. **c**, Barplot exhibiting the number of cells that can be assigned to a specific cluster for each of the individual capture areas within the integrated spatial dataset.

To identify genes associated with AM fungi colonization within our spatial datasets, we performed dimensionality reduction and clustering of all voxel transcriptome profiles from both AM fungi- and mock-inoculated spatial capture areas (Extended Data Fig. 2). Due to the relatively low resolution of the Visium platform (each 55-μm voxel could contain one to five cells), we refrained from assigning cell identities to spatial dataset clusters (referred to as ‘spatial clusters’), as voxels probably represent heterogeneous cell groups. Instead, we identified voxel clusters within the mycorrhizal dataset that could represent sites of AM colonization. To do so, we compiled a list of established AM-responsive marker *M. truncatula* and *R. irregularis* genes (Supplementary Table 3) functioning in various stages of AM colonization and analysed their expression across the 13 spatial clusters in reference to a stably expressed housekeeping gene, elongation factor 1 alpha (*MtEF1α*)^40^. Spatial clusters 3 and 12 showed high specific expression of the markers from both species and thus were deemed ‘AM responsive’ (Fig. 3b), with spatial cluster 12 showing higher expression of early markers and spatial cluster 3 showing higher expression of late-stage markers. We detected a few AM symbiosis marker genes within the snRNA-seq dataset that were missing or lowly expressed within the spatial datasets. This may be due to lower detection efficiency of unbiased transcriptomic methods as compared with probe-based capture^41^. To estimate spatial expression of these genes, we integrated nuclear and spatial datasets together and imputed expression values across modalities. Using this approach, we associated the expression of two marker genes, does not make infection 1 (*MtDMI1*)^42^ and sickle 1 (*MtSKl1*)^43^, with mycorrhizal capture areas, despite their absence from the spatial dataset.

### Colonization stage-specific *M. truncatula* gene expression

**Fig. 4:**
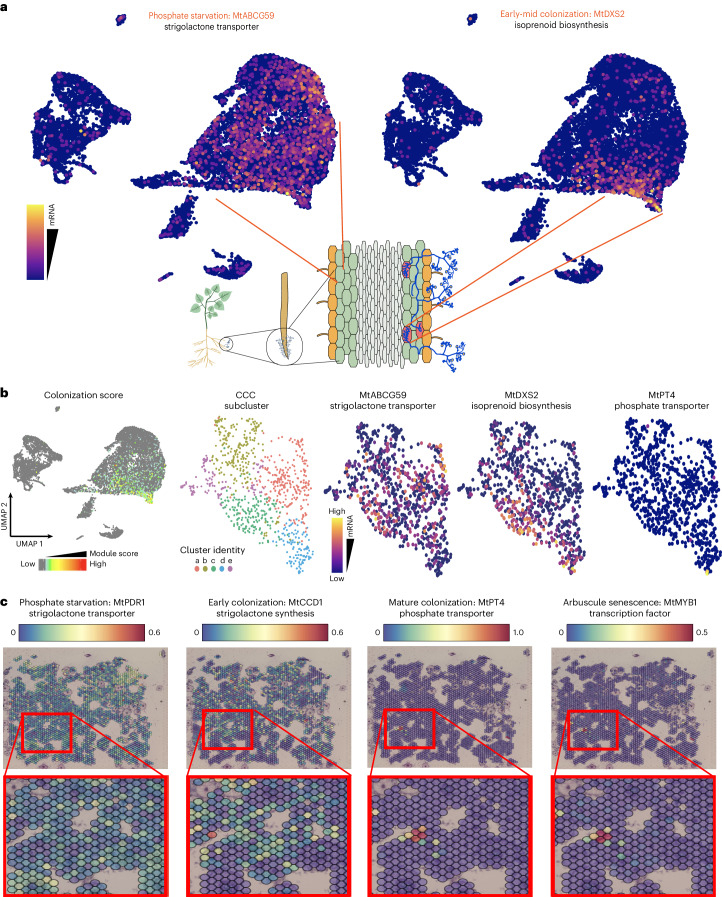
Analysing colonization stage-specific gene expression. **a**, The expression of canonical pre- and post-AM marker genes shown in a UMAP plot of single-nuclei dataset, coloured by normalized mRNA counts (scale, log(UMI counts + 1)). **b**, Module scores based on average expression level of a list of known AM marker genes; cells with module scores in the 98th percentile were selected as ‘CCCs’ and clustering was performed on this subset. Three AM marker genes known to be expressed at different colonization stages are expressed in different subclusters of the colonized cluster. **c**, Spatial feature plots of four AM marker genes known to be expressed at different colonization stages: *MtPDR1*, *MtCCD1*, *MtPT4* and *MtMYB1*. Zoom in red blocks focuses on voxels that switch expression profiles.

We used stage-specific markers to determine the distribution of cells across arbuscule development within the snRNA-seq datasets. Low phosphate availability stimulates the interaction between plant and AM fungus, resulting in secretion of strigolactones from cortex cells^44^. ABCG transporter 59 (*MtABCG59*) is upregulated during phosphate starvation and upon mycorrhizal exposure^45^. We observed enrichment of *MtABCG59* in cells throughout the cortex clusters (Fig. 4a), representing cells that are responding to phosphate starvation. Cluster 14, which specifically expressed 1-deoxy-d-xylulose 5-phosphate synthase (*MtDXS2*) transcripts, probably represents cells undergoing active AM symbiosis, as *MtDXS2* is required for the methyl d-erythritol phosphate pathway-based isoprenoid production to sustain AM colonization (Fig. 4a)^46^. To determine whether we captured a developmental gradient, we first defined an expression module of AM symbiosis marker genes expressed in the AM symbiosis cluster (cluster 14), and then we computed an AM module score for all cells (Methods). We selected cells in the 98th percentile for this AM module score and re-clustered them into five subclusters (Fig. 4b). Based on the enrichment of marker genes observed, subclusters a, b and e probably represent earlier stages, as these cells are enriched for *MtABCG59* transcripts. Clusters c and d may represent later stages of colonization based on the enrichment of *MtDXS2* transcripts, and the cells at the edge of cluster d are probably at the most advanced stage of colonization, as they are the only cells in the single-cell datasets that contain high levels of *MtPT4* mRNA (Fig. 4b).

**Fig. 5:**
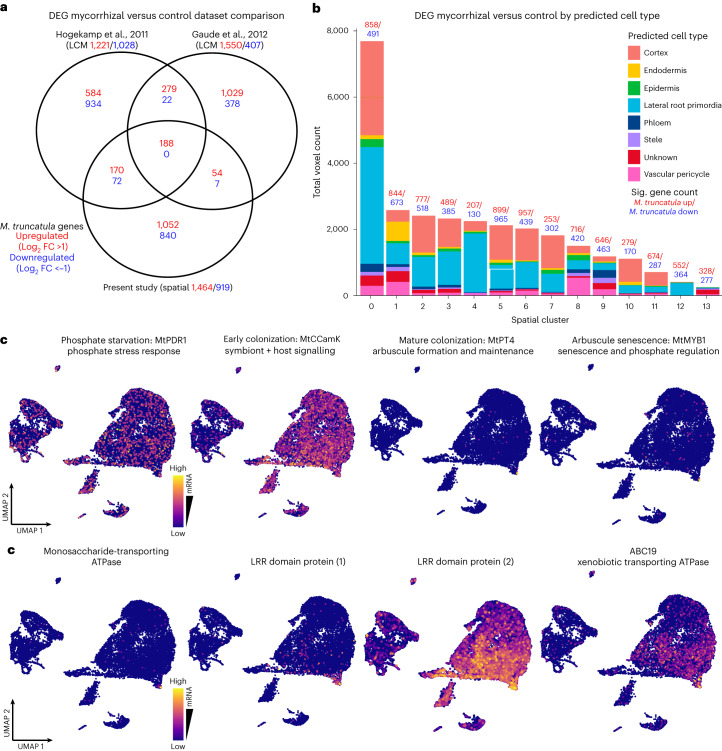
Existing and novel transcriptomic studies reveal a robust set of differentially expressed *M. truncatula* genes during the AM symbiosis. **a**, A Venn diagram showing overlap in symbiosis-responsive *M. truncatula* genes with a log fold change > or <1 between the spatial dataset from this study and the two previously published LCM RNA-seq studies from Gaude et al.^15^ and Hogekamp et al.^14^. **b**, The breakdown of predicted cell types represented in each cluster within the integrated spatial dataset in terms of number of voxels. DEGs between mycorrhizal and control treatments are shown above each bar for each cluster, with the counts of significantly (Sig; see Methods) upregulated *M. truncatula* genes in red and downregulated gene counts in blue. **c**, The expression of four canonical pre- and post-AM symbiosis marker genes shown in a UMAP plot of single-nuclei dataset (upper). The expression of four genes enriched in the CCC cluster of this dataset that have yet unknown roles in AMF colonization, shown in a UMAP plot of single-nuclei dataset (lower). The UMAP plots are coloured by normalized mRNA counts (scale, log(UMI counts + 1)) depending on the marker gene, thus reflecting the colonization stage.

We also visualized the spatial dynamics of colonization by tracking the distribution of stage-specific AM symbiosis marker genes. By analysing expression of *MtABCG59*, carotenoid cleavage dioxygenase 1 (*MtCCD1*)^47^, *MtPT4* and MYB-like transcription factor 1 (*MtMYB1*)^48^, we classified voxels within distinct stages of colonization (Fig. 4c). We repeated this stage-specific analysis with the snRNA-seq data to show the developmental trajectory of AM colonization starting with the phosphate stress response gene pleiotropic drug resistance 1 (*MtPDR1*)^49^, which is enriched throughout the cortex cluster. Calcium and Ca^2+^/calmodulin-dependent protein kinase (*MtCCaMK*)^50^ is involved in host–symbiont signalling in early stages and is enriched in the cells closer to the colonized cluster. *MtPT4* is enriched in a subset of cells at the furthest edge of the colonized cluster and *MtMYB1* is also enriched in these distal cells (Fig. 5c, upper panel).

**Extended Data Fig. 3:**
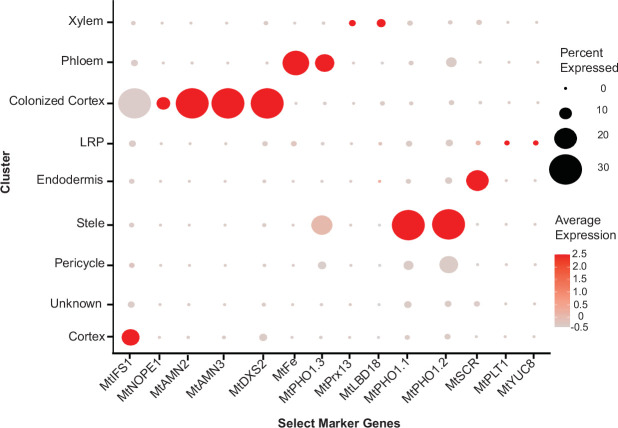
Lipid transfer gene expression for single-nuclei clusters. Dot-plot of expression profiles for *M. truncatula* genes involved in lipid transfer to the fungal symbiont across labeled cluster identities utilizing hierarchical clustering within the integrated single-nuclei datasets.

Performing differential gene expression analysis, we found 258 genes enriched in the AM symbiosis cluster 14 (log FC >0.25, adjusted *P* < 0.01, Supplementary Table 4) compared with all other cortex clusters combined. Among these, we recovered known marker genes for AM symbiosis, along with many genes not previously associated with AM signalling as well as genes with no annotated function. These included a gene encoding a monosaccharide transporting ATPase (*Medtr8g006790/MtrunA17_Chr8g0335291*), which we speculate the plant uses to provide sugars to the fungus, several genes with leucine-rich repeat domains (*Medtr6g037750/ MtrunA17_Chr6g0464631* and *Medtr3g058840/MtrunA17_Chr3g0102261*), which could mediate host–symbiont signalling, and *MtABC19*, which encodes a xenobiotic-transporting ATPase (*Medtr3g093430/ MtrunA17_Chr3g0128391*) (Fig. 5c, lower panel). Lastly, we observed high expression of several lipid transfer *M. truncatula* genes within this cluster (Extended Data Fig. 3).

## A robust set of symbiosis-responsive *M. truncatul*a genes

Differentially expressed genes (DEGs) between mycorrhizal and control spatial capture areas revealed 2,383 AM-responsive *M. truncatula* transcripts (Fig. 5a). Of these, 1,464 were upregulated (log_2_ FC >1.0) and 919 were downregulated (log_2_ FC <1.0) in the mycorrhizal treatment (Supplementary Table 5). Two LCM-based transcriptomic analyses revealed similar numbers of DEGs in response to mycorrhizal treatment^24,25^, with 188 genes significantly upregulated across all three datasets, which we refer to as ‘robust’ AM-responsive genes (Fig. 5a and Supplementary Table 5). No genes were found to be significantly downregulated in all three datasets. This set of robust AM-responsive genes includes characterized AM symbiosis marker genes, such as *MtMYB1*, *MtPT4* and *MtRAD1* (a positive GRAS transcription regulator of the symbiosis)^51^. One of the upregulated transcripts cyclin-like 1 (*MtCYC1*) encodes a putative cyclin-like F-box protein and is a known marker for cell division^52^, suggesting induction of cortical cell division as a response to AM colonization. However, many of the 188 genes remain to be investigated for their role in AM symbiosis.

The 55-μm resolution of the Visium Spatial Gene Expression platform results in blending of several adjacent cells together into a single profile (that is, it cannot resolve transcripts into single cells). However, we utilized our annotated snRNA-seq and spatial datasets to predict the proportions of each cell type represented in each spatial cluster (Fig. 5b). Differences were observed in the proportion of cell types between spatial clusters. We saw a relatively high proportion of voxels identifying as lateral root primordia, which we hypothesize as resulting from the high amount of mRNA captured from meristematic root tissue on the capture area. We also observed large differences in the amount of DEGs between the individual spatial clusters, indicating the method can discern between cell populations experiencing different degrees of AM colonization.

### Functional enrichment depicts a marked symbiotic response

**Extended Data Fig. 4:**
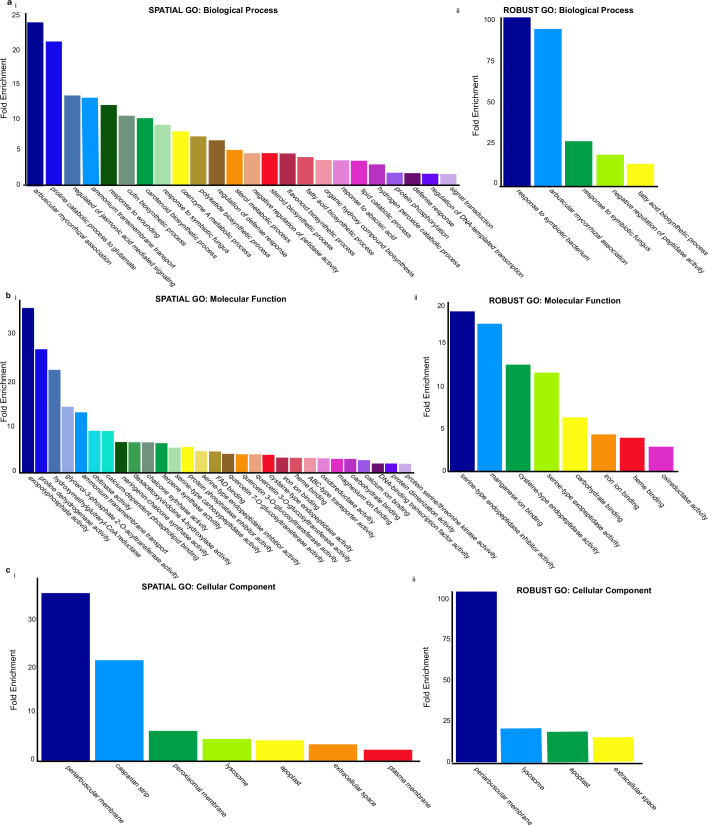
Gene-set enrichment analysis reveals AM-specific gene functionalities within the mycorrhizal spatial datasets and robust gene list. Gene ontology enrichment scores for the main biological processes **(a)**, molecular functions **(b)**, and cellular components **(c**) represented within the significantly upregulated genes between the mycorrhizal and control treated i) spatial datasets and ii) robust gene list as compared to the reference genome.

We performed functional enrichment analysis for Gene Ontology terms, including biological process (Extended Data Fig. 4a), molecular function (Extended Data Fig. 4b) and cellular component (Extended Data Fig. 4c) among significantly upregulated *M. truncatula* genes (I, Supplementary Tables 6 and 7) and the robust gene set (II, Supplementary Tables 8 and 7). As expected, we saw a >20-fold enrichment for the ‘arbuscular mycorrhizal association’ biological process term overall, and >80-fold enrichment for this term within the robust set. We also observed a high enrichment (>100-fold) of the ‘response to symbiotic bacterium’ term within the robust set, indicative of genes common to both fungal and bacterial (rhizobial) symbioses. During rhizobial symbiosis, legumes allow controlled infection by rhizobia, leading to the development of root nodules in which rhizobia directly fix and transfer nitrogen to their hosts. The rhizobial symbiosis coopted numerous components of the ancestral AM symbiosis signalling mechanism^53^ and this ‘common symbiotic signalling pathway’ is represented within our robust dataset by the differential expression of two co-expressed interacting genes, MtVAPYRIN (*MtVPY*)^54^ and MtEXOCYST70 (*MtEXO70i*)^55^. These genes function during the intracellular phases of endosymbiosis in both AM and rhizobial symbioses, with *MtVPY* genetic mutants impaired in arbuscule and infection thread development, respectively^56^. In the AM symbiosis, *MtVPY* interacts with *MtEXO70i*, which is critical to PAM development and arbuscule branching^57^. Another robust gene, cysteine protease 3 (*MtCP3*), probably plays a role in arbuscule degeneration, functioning to degrade the PAM^48^, and also contributes to nodule senescence^58^, indicating a common functionality. Interestingly, the biological process ‘proline catabolic process to glutamate’ and the molecular function ‘proline dehydrogenase’ showed a >20-fold enrichment (Extended Data Fig. 4a,b). Several studies noted altered proline levels under drought stress in AM-treated plants^59,60^, and hypothesized that proline confers protection from changes in water availability. As we did not apply a drought stress to plants in our survey, we believe proline metabolism may contribute to a different protective mechanism.

AM fungi convert soil inorganic phosphate (Pi) into inorganic polyphosphate (polyP) and can rapidly accumulate and translocate polyP within hyphae^61^. AM fungi also depolymerize polyP via fungal endopolyphosphatases and transfer this phosphorus into host plant cells across the PAM^62^, although the mechanism for this export remains unclear. Some evidence suggests that the majority of this export occurs via the transport of Pi across the apoplastic space and subsequent uptake by the plant via Pi transporters^62^. However, a growing amount of evidence suggests polyP may be directly exported to the apoplastic space and then hydrolysed by the plant itself^61^. Nguyen and Saito provided evidence that fungal-derived polyP and plant-derived phosphatases had opposing localizations in mature arbuscules, indicating that plant phosphatase activity could account for assimilation of fungal-derived polyP^63^. Surprisingly, ‘exopolyphosphatase activity’ exhibited the highest enrichment of all molecular functions, (>30-fold) in our dataset. This adds support to the hypothesis that polyphosphatase activity by the plant plays a larger role in phosphorus export than previously anticipated.

Lastly, we expected that cellular components enriched in our datasets would include those involved in AM symbiosis, such as the PAM and the plant plasma membrane. The robust dataset showed a >100-fold enrichment for the ‘PAM’ category and captured 100% of the *M. truncatula* genes assigned to that functional category within the genome (Extended Data Fig. 4c). Overall, functional enrichment analysis confirmed a strong symbiotic signature within our mycorrhizal dataset.

### Novel symbiosis-responsive *R. irregularis* gene expression

**Fig. 6:**
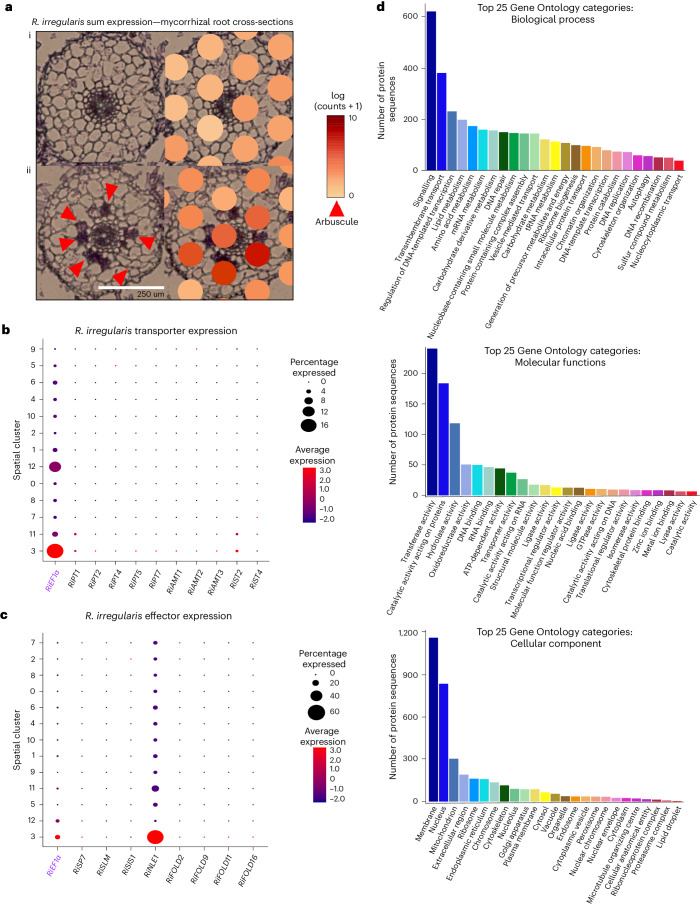
Spatially resolved *R. irregularis* transcripts reveal novel AM-specific gene expression patterns. **a**, A single root cross-section within capture area (left) and overlapping distributions of all *R. irregularis* transcript expression (right) visualized in Loupe Browser. i, A representative root cross-section from a mycorrhizal capture area lacking recognizable fungal structures shows low expression of *R. irregularis* transcripts. ii, A representative root cross-section from a mycorrhizal capture area that contains visible arbuscules (red arrows) shows high expression of *R. irregularis* transcripts, particularly around arbuscules (scale bar, 250 µm; scale, log(UMI counts + 1)). Approximately 50 root cross-sections across nine mycorrhizal-treated capture areas were analysed. **b**, A dot plot of RiEF1α and various transporters utilizing hierarchical clustering within integrated mycorrhizal spatial object. **c**, A dot plot of RiEF1α and various effector proteins utilizing hierarchical clustering within the integrated mycorrhizal spatial object. **d**, A bar plot depicting top 25 Gene Ontology categories for all expressed *R. irregularis* transcripts (biological process (i), molecular function (ii) and cellular component (iii)).

Bulk^64–68^ and LCM-based single-cell^24,25^ RNA-seq studies conducted on *M. truncatula* in symbiosis with *R. irregularis* utilized a broad spectrum of cell isolation and transcriptomic techniques. In addition, considerable progress has recently been made^68–73^ to build our knowledge of the genetic landscape of AM fungi and fungal gene expression occurring within this interaction. However, simultaneous capture of plant and fungal mRNA during symbiosis remains challenging. Our approach built upon existing research by providing the first spatially resolved dataset of simultaneously captured plant and fungal transcriptomes during the AM symbiosis. We detected expression of 12,104 unique fungal transcripts across nine mycorrhizal capture areas (Supplementary Table 9). Fungal gene expression distribution across the capture areas overlaps arbuscules in the tissue (Fig. 6a). Voxels spanning root cross-sections that display a high degree of arbusculation also exhibit high expression of total *R. irregularis* transcripts. AM fungi provide their hosts with hard-to-access soil nutrients through the actions of transporters across the PAM^74^ and benefit from the continuous transfer of lipids and sugars from host plant to fungus^75^. We observed the localized expression of five phosphate transporters (*PT1[RIR_1575600/g11592]*, *PT2[RIR_1235500/g7615]*, *PT4[RIR_0355700/g31083]*, *PT5[RIR_3213400/g18438]* and *PT7[RIR_2900800/g19437]*), three ammonium transporters (*AMT1[RIR_0149600/g16666]*, *AMT2[RIR_0697800/g1222]* and *AMT3[RIR_0390200/g18142]*), and two sugar transporters (*ST2[RIR_2811400/g24501]* and *ST4[RIR_0496600/g26862]*) to spatial cluster 3, the cluster identified as symbiosis-responsive via localization of *M. truncatula* AM symbiosis markers. We observed lower expression of transporter transcripts relative to that of *RiEF1α*, although we did observe expression of *RiPT1*, *RiPT4*, *RiAMT1*, *RiAMT2* and *RiST2*. Spatial cluster 3 showed localized expression of *M. truncatula* transporters, such as *MtPT4*, *MtAMT2* and *MtSWEET1b*^76^ (Fig. 3b), indicative of active nutrient transport occurring in both partners in these voxels.

The same analysis was performed for eight fungal effectors (Fig. 6c), including secreted protein 7—RIR_3212100/g18424 (*RiSP7)*, an effector protein involved in biotrophic response to AM colonization^77^, secreted lysin motif—RIR_1359320 (*RiSLM*), an effector which reduces chitin-triggered immune responses during symbiosis^78^, SL-induced putative secreted protein 1—RIR_2427800/g2579 (*RiSIS1*), a secreted protein induced in both AM pre-symbiotic and symbiotic phases^69^, and nucleus localized effector 1—RIR_2535800/g7021 (*RiNLE1*), an effector upregulated in arbuscules involved in the suppression of defence responses^79^, as well as four members of the MycFOLD effector family (*RiMycFOLD2*—RIR_3103500/g17566, *RiMycFOLD9*—RIR_2782800/g17548, *RiMycFOLD11*—RIR_098400/g17317, and *RiMycFOLD16*—RIR_1383400/g25859)^80^. Most effectors exhibited specific expression in spatial cluster #3, though relative to *RiEF1α*, the expression of one of these effectors, *RiNLE1*, was extremely high (Fig. 6c). In fact, *RiNLE1* represented the fifth most highly expressed fungal transcript. Evidence suggests that *RiNLE1* translocates to the plant nucleus of arbusculated cells and interacts with the histone H2B protein to suppress defence responses via epigenetic modification of *MtH2B* (*Medtr4g064020/MtrunA17_Chr4g0031671*)^79^. Altogether, the high level of expression and high proportion of cells (60%) that *RiNLE1* is expressed in within spatial cluster 3 further supports that this spatial cluster represents arbusculated cells and that other marker genes that specify this cluster are probably involved in AM symbiosis. Supplementary Table 10 describes all plant and fungal transcripts found to be expressed in spatial cluster 3.

Lastly, we identified at least one Gene Ontology classification for 8,559 out of the 12,104 expressed transcripts (Supplementary Table 11). The top biological processes represented include ‘signalling’, ‘transmembrane transport’ and ‘lipid metabolism’, consistent with processes carried out during AM symbiosis (Fig. 6d, i). Similarly, ‘transferase activity’, ‘transporter activity’ and ‘lipid binding’ rank highly among molecular function terms (Fig. 6d, ii). ‘Membrane ‘and ‘nucleus’ were well represented among cellular components, with >1,000 and >800 transcripts classified to each, respectively (Fig. 6d, iii). This dataset of >12,000 symbiosis-responsive spatially resolved *R. irregularis* genes and corresponding tissue images is the first of its kind and holds immense potential for in-depth characterization.

## Discussion

Advances in single-cell transcriptomics transformed molecular genetics by allowing cell type-specific analysis and, more recently, conservation of the tissue context^81^. Here we combined single-nucleus and spatial RNA-seq to construct the first high-resolution spatially resolved integrated map of a multi-kingdom symbiotic interaction. We successfully adapted the spatial transcriptomics platform for use with plant roots and utilized transcriptome-wide mRNA capture from two species simultaneously. In addition, we identified cell type-specific responses to the AM symbiosis and integrated data from both approaches to discover novel AM-responsive transcripts.

snRNA-seq increases resolution and throughput for the identification of cell-state responses to external treatments and eliminates the hurdle of protoplasting cells^82^. snRNA-seq provides a potential advantage over scRNA-seq in conceptually allowing profiling of plant and fungal nuclei in the same assay. However, even in batches of nuclei not subjected to gated flow sorting, we failed to recover a substantial number of fungal transcripts and obtained no defined fungal nuclei, possibly because fungal nuclei were not captured or were destroyed by our plant nuclei extraction protocol. AM fungi consist of multinucleate hyphae in a large syncytium but differ from other multinucleated fungi due to the unusually high number of nuclei (up to 35,000) that exist within their cells^83^. This aspect of their biology may complicate interpretation of snRNA-seq data when compared with spatial or bulk RNA-seq.

Spatial transcriptomics allows for a side-by-side comparison of gene expression and tissue features and enabled the capture of fungal transcripts. Yet limitations exist for this technology as well, notably a super-cellular resolution (large inter-voxel distances that miss many cells and large voxel sizes that blend adjacent cells together). Probe-based capture technologies improve the resolution of spatial transcriptomics, but this limits analysis to a set of pre-defined genes, and may also lead to lower capture efficiency due to fewer primers^84^. There is a clear need for a high-resolution spatial technology that allows for unbiased mRNA capture from intact tissue.

We found 258 *M. truncatula* genes that were upregulated in the CCCs versus all other cortex cells of mycorrhizal-treated snRNA-seq datasets. These genes were enriched for Gene Ontology terms related to fungal symbiosis, terpene synthesis pathways and transport proteins. We also found 17 different cytochrome P450-like proteins, whose roles in plant–microbe interactions include hydroxylation of fatty acids and terpene synthesis^85^, and 19 leucine-rich repeat domain-containing proteins that are typically associated with pathogenesis but have been shown to also be upregulated in AM symbiosis^85,86^. Several xenobiotic, sugar and amino acid transporters were also upregulated in CCC. The identification of known and novel transcripts within the CCC cluster, along with the 188 genes upregulated among three distinct RNA-seq studies in the same symbiotic system, presents a community resource for characterization of novel AM-associated genes.

*R. irregularis* forms symbioses with many diverse plant species in natural and agricultural ecosystems^87^ and serves as a model species for the AM symbiosis. Despite the biological importance of this fungus, functional annotation and characterization of its genes has been slow due to the difficulty in its genetic manipulation^88^. Recent advances in CRISPR-Cas9^89^ gene editing and continued efforts in creating pure cultures of AM fungi^18,19^ will hopefully bring forth a new chapter of AM symbiotic research that focuses on the fungal partner. Recent efforts in improving AM fungal genome assemblies^90,91^ and functional characterization of *R. irregularis* genes involved in symbiosis^69,77–79,92,93^ lay the groundwork for this shift. We identified spatial cluster 3 as AM-responsive based on the expression of both *M. truncatula* and *R. irregularis* marker genes for the AM symbiosis. The transcripts expressed within this cluster, as well as the thousands of *R. irregularis* genes expressed within the mycorrhizal capture areas in this study, represent excellent targets for functional characterization studies in both partners and it is our hope that the AM community can use these datasets as a resource to uncover new functionalities.

## Methods

### Plant growth and inoculation

Seeds of *Medicago truncatula* Gaertn, cv Jemalong A17 (Noble Foundation) were scarified in concentrated sulfuric acid for 5–10 min and rinsed with distilled water, sterilized in 3.75% sodium hypochlorite solution, then rinsed five times with sterile distilled water and placed on 1∕2 Murashige and Skoog medium, 1% agar plates at 4 °C or room temperature for 48 h. Sand cones were prepared as follows: 8.25-inch cone-tainers (Stuewe and Sons) with 1 cm^3^ rock wool at base, filled up to 12.7 cm with autoclaved calcined clay (Turface Athletics MVP 50), followed by 2.5 cm of autoclaved horticultural sand (American Soil and Stone) and topped with 2.5 cm fine play sand (SAKRETE). To inoculate seedlings with *Rhizophagus irregularis* (Błaszk., Wubet, Renker and Buscot) C. Walker and A. Schüßler, 50 ml Agtiv Field Crops liquid mycorrhizal inoculant (PremierTech) spores were captured on a 40-µm filter, rinsed with distilled water and resuspended in 50 ml distilled water. A total of 1 ml of resuspended spores was applied to the horticultural sand layer and an additional 300 µl were applied to the fine play sand layer. Germinated seedlings were transplanted to the top fine sand layer of inoculated and non-inoculated sand cones. Plants were grown in 22–24 °C with 16 h day/8 h night, with 300 μmol m^−2^ s^−1^ light intensity and 60% relative humidity. Plants were watered daily and fertilized twice a week with 1/2× Hoagland’s medium modified with 20 μM phosphate to stimulate AM colonization.

### Colonization assessment

**Extended Data Fig. 5:**
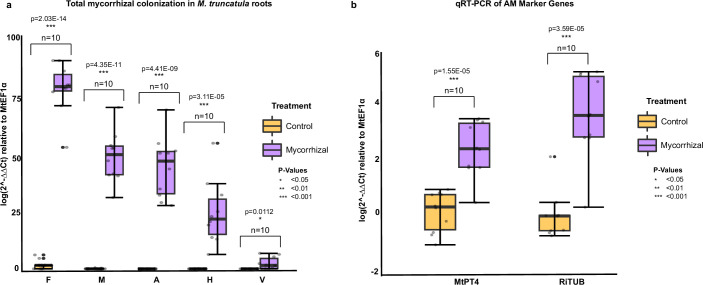
Root AM colonization analyses. **a**, AM colonization quantified by the Trouvelot method at 28 dpi with *R. irregularis* in roots; Data are presented as mean values +/− SEM (n = 10). F%, frequency of infection; M%, total mycorrhization, A%, total arbuscule abundance; H%, total intraradical hyphae abundance, V%, total vesicle abundance. *n* represents the number of biologically independent samples. P values were determined using one-way analysis of variance (ANOVA) (* indicates p-value <0.05, ** p<0.10, ***.001). **b**, Expression of MtPT4 and RiTUB relative to MtEF1-a measured by qRT-PCR; Data are presented as mean values +/− SEM (n = 10). The box plots in a and b indicate the median (line inside the box), the lower and upper quartiles (box), margined by the smallest and largest data within the interval of 1.5x the interquartile range from the box (whiskers); outliers are shown (data points outside of box). n represents the number of biologically independent samples. P values were determined using one-way analysis of variance (ANOVA)(* indicates p-value <0.05, ** p<0.10, ***.001).

AM colonization in wild-type roots at 21, 28 or 38 dpi was visualized via staining of AM chitin using 2.5 μg ml^−1^ wheat germ agglutinin Alexa Fluor 488 (Thermo Fisher Scientific) in 1× phosphate-buffered saline (PBS) solution (pH 7.0). Briefly, roots collected from the fine sand layer were rinsed, fixed in 50% ethanol for 30 min and cleared in 10% KOH at 65 °C for 48 h. Cleared roots were neutralized with 0.1 M HCl and stained with wheat germ agglutinin 488 in 1× PBS at 4 °C for 24 h before imaging. Colonization was quantified using the Trouvelot method^94^ on a Leica DM6B fluorescence microscope using five biological replicates for each treatment (Extended Data Fig. 5a and Supplementary Table 12).

### Quantitative real-time PCR of target genes

To quantify expression of target genes, 100 mg of roots from the fine sand layer were flash frozen in liquid nitrogen. Total RNA was extracted using the RNeasy Plant Mini Kit (Qiagen) and corresponding DNase. Complementary DNA synthesis was conducted using the SuperScript IV Reverse Transcriptase (Thermo Fisher Scientific) from 500 ng of total RNA, and quantiative polymerase chain reaction (qPCR) was conducted from cDNA diluted 1:5 using the PowerUp SYBR Green Master Mix (Thermo Fisher Scientific). A 200 nM primer concentration and the following protocol were used for qPCR for all targets: 2 min at 50 °C and 2 min at 95 °C, followed by 39 repeats of 15 s at 95 °C, 15 s at 60 °C and 1 min at 72 °C, and ending with 5 s at 95 °C. A melting curve (55–95 °C; at increments of 0.5 °C) was generated to verify the specificity of primer amplification. Five biological replicates and three technical replicates of all targets (*MtPT4* and *RiTUB*) were quantified for gene expression levels relative to the housekeeping gene *MtEF1α* using the ΔΔCT method (Extended Data Fig. 5b). All primer sequences used for qPCR can be found in Supplementary Table 13. Raw ΔΔCT values used for statistical analysis can be found in Supplementary Table 14.

### Nuclei and bulk root tissue RNA profiling

*M. truncatula* roots from three plants from each treatment were harvested at 21, 28 and 38 days post-inoculation; 150 mg of roots grown in the inoculated fine sand layer was either flash frozen in liquid nitrogen, or nuclei extraction was performed up to the 20 µm filtration step and then flash frozen. RNA was extracted using the RNeasy Plant Mini Kit (Qiagen). Library preparation and sequencing were performed at the QB3 UC Berkeley Genomics Core Sequencing Facility.

### Nuclei extraction and sequencing

*M. truncatula* roots from three plants per condition were harvested at 21, 28 or 38 dpi. A total of 150 mg of roots growing in inoculated fine sand layer was weighed out and placed in the lid of a petri dish and chopped rapidly with a razor blade for 3 min in 600 µl NIBAM (1x NIB, Sigma CELLYTPN1-1KT; 4% BSA; 1 mM DTT; 0.4 U µl^−1^ Superase RNAse inhibitor, Sigma; 1:100 Protease Inhibitor Cocktail for plant tissues, Sigma). NIBAM root slurry was strained through 40 µm and 20 µm filters (CellTrics). SYBR Green (1:10,000) was used to visualize nuclei during purification on the Influx Flow Cytometer. A total of 20,000 nuclei were sorted into 19 µl of ‘landing buffer’ (PBS with 0.4 U µl^−1^ Superase RNAse inhibitor) with a final volume of 43 µl. DAPI was applied to 2 µl of nuclei suspension to evaluate the quality of nuclei on a Leica AxioObserver at 20× magnification. The remaining 41 µl was mixed with 10× Genomics Chromium RT Master Mix with no additional water added and loaded onto a Chromium Chip G, and thereafter the standard manufacturer’s protocol was followed (V3.1 Dual Index). Twelve cycles were used for cDNA amplification, and the completed cDNA library was quantified using an Agilent Bioanalyzer. Sequencing was performed at the QB3 UC Berkeley Genomics Core Sequencing Facility on a single NovaSeq SP lane with the sequencing parameters 28 bp (read 1 length), 10 bp (index 1 length), 10 bp (index 2 length) and 90 bp (read 2 length), or at Novogene (Sacramento, CA) using the sequencing parameters 150 bp (read 1 length), 10 bp (index 1), 10 bp (index 2) and 150 bp (read 2 length).

### Tissue preparation for spatial transcriptomics

**Extended Data Fig. 6:**
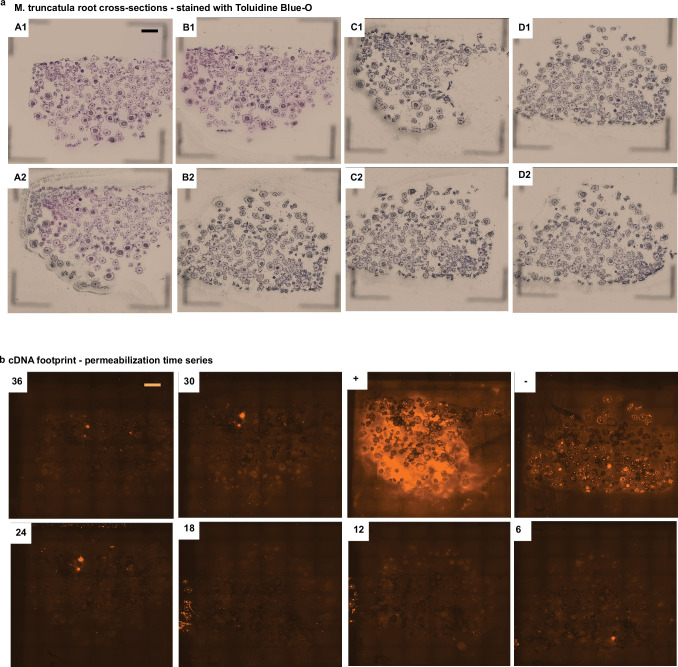
Visium tissue optimization. **a**, Brightfield images of eight capture areas on Visium Tissue Optimization slide containing 16 $\mu m$- thick *M. truncatula* lateral root cryo-sections after methanol fixation and staining with Toluidine Blue-O (scale bar = 1mm). Experiment was repeated twice, for a total of 16 capture areas. **b**, Corresponding cDNA footprint after fluorescently-tagged nucleotides were incorporated into reverse transcription reaction across 6 different permeabilization timepoints (scale bar = 1mm). Timepoint of 12 minutes was chosen for subsequent Visium Gene Expression workflows.

Spatial transcriptomics was performed with the Visium Spatial Gene Expression platform from 10x Genomics. Harvested plant roots were rinsed with deionized H_2_O and cryopreserved in optimal cutting temperature compound via submerging of optimal cutting temperature-embedded molds into a dewar of isopentane chilled liquid nitrogen for even freezing. Cryomolds of roots were stored at −80 °C until cryosectioning. Cryosectioning was performed on an Epredia CryoStar NX70 Cryostat with a blade temperature of 14 °C and a sample head temperature of −12 °C with a section thickness of 16 °C. Cryosections were placed onto the surface of the chilled Visium Spatial Gene Expression slide and adhered to the slide using heat from the sectioner’s finger placed on the back surface of the capture area. Prepared slides were stored at 80 °C before processing for 10× Visium spatial transcriptome sequencing according to the manufacturer’s instructions with the following modifications: first, cryosections were stained using an incubation of 0.05% toluidine blue O in 1× PBS for 1 min and rinsed three times with 1× PBS. Second, a pre-permeabilization step was added as suggested by Giacomello et al.^95^. The pre-permeabilization mix for each slide (48 µl exonuclease I, 4.5 µl of bovine serum albumin and 428 µl of 2% PVP 40) was then prepared, and 70 µl was pipetted into each well. Pre-permeabilization occurred for 30 min at 37 °C after which the manufacturer’s protocol for tissue permeabilization was followed. Permeabilization enzyme (70 µl) was added to each capture area and incubation at 37 °C occurred for 12 min on the basis of the results of the manufacturer’s tissue optimization protocol (Extended Data Fig. 6).

### Data processing and analysis

Cellranger and Spaceranger software (10x Genomics) were used to preprocess single-nuclei and spatial transcriptomic sequencing libraries, respectively. A formatted reference genome was generated using Cellranger or Spaceranger’s ‘mkref’ function using the *Medicago truncatula* MedtrA17_4.0 (ref. ^37^) whole genome sequence and annotation and the *Rhizophagus irregularis* Rir_HGAP_ii_V2 (DAOM 181602, DAOM 197198)^38^ whole genome sequence and annotation using default parameters. Single-nuclei and spatial reads were aligned to the genome references using the ‘count’ function in Cellranger 7.0 and Spaceranger 1.3 software packages (10x Genomics), respectively. Brightfield tissue images were aligned to the spatial capture area fiducial frame and voxels corresponding to overlaying tissue were manually selected for all capture areas in Loupe Browser (10x Genomics). Data analysis for both the single-nuclei and spatial data was performed using the Seurat^22^ 4.3.0 package in R 4.2.1 available at https://www.R-project.org.

#### Filtering and normalization

For both single-nuclei and spatial datasets, normalization and scaling were performed using the SCTransform R function in Seurat before clustering. Metrics used for filtering of the data during quality control steps can be found in Supplementary Table 2.

#### Principal components analysis and *K*-means clustering

**Extended Data Fig. 7:**
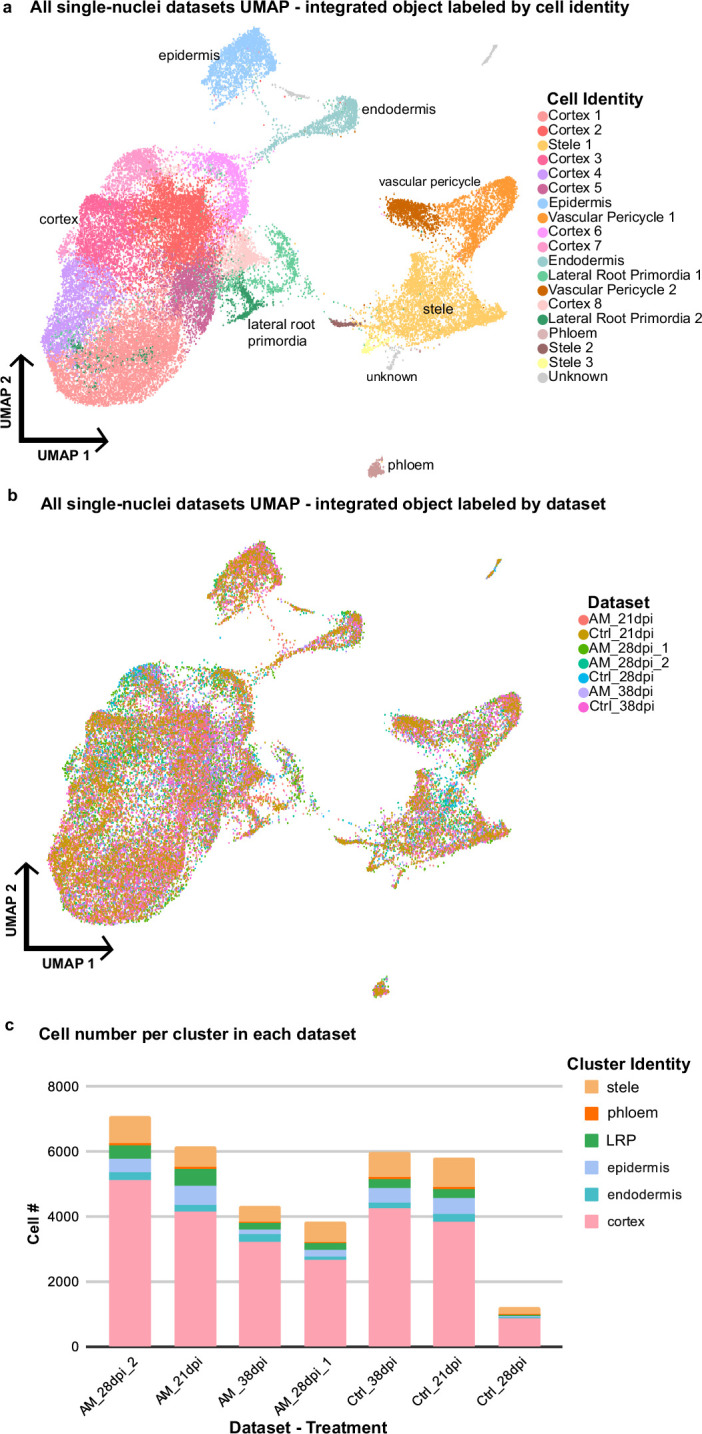
Single-nuclei RNA-seq integrated UMAPs by treatment and dataset. **a**, UMAP of 38,096 M*. truncatula* nuclei from the integrated Seurat object of all root harvests. Identities of 19 unique clusters are represented by different colors according to cell type. Clusters without a known identity are marked as ‘unknown’. **b**, UMAP of 38,096 M*. truncatula* nuclei from the integrated Seurat object of all root harvests. Different colors correspond to the original dataset. **c**, Barplot exhibiting the number of cells that can be assigned to a specific cell type identity for each of the individual datasets within the integrated single nuclei dataset.

Principal components analysis was performed on both snRNA-seq and spatial RNA-seq datasets using the RunPCA function in Seurat with the ‘SCT’ assay specified. The FindNeighbors function was applied to construct a shared nearest neighbour graph for the data using the first 30 principal components. Clustering was performed using the FindClusters function, which utilizes the shared nearest neighbour graph from the previous step. Finally, the RunUMAP function was utilized to construct the uniform manifold approximation and projection (UMAP) dimensionality reduction and visualize the dataset in two dimensions. Of the seven snRNA-seq datasets generated (Extended Data Fig. 7 and Supplementary Table 2), we selected five for further characterization, resulting in a final dataset of 16,890 nuclei with an average of 1,120 mRNA molecules per cell after quality filtering. The remaining two datasets were not included due to poor apparent colonization by *R. irregularis*. All spatial datasets were analysed.

#### Integration of replicate datasets

Replicate capture areas or samples from each treatment (mycorrhizal or mock-inoculated) were integrated into a two sets of Seurat objects (snRNA-seq, spatial) using the data integration pipeline in Seurat^96^. First, we applied the PrepSCTIntegration function using all transcripts for integration. We then identified a set of integration anchors with the FindIntegrationAnchors function. Finally, we applied the IntegrateData function. Principal component analysis and dimensionality reduction were performed on the integrated objects in the same manner as the individual objects with the following adjustments: (1) number of principal components, 30; (2) metric, cosine; and (3) resolution was set to 0.5 for clustering. UMAP plots for all datasets were created using the DimPlot() function and are displayed in Extended Data Fig. 7 (snRNA-seq) and Extended Data Fig. 2 (spatial).

### Cluster identification for snRNA-seq

We determined that clusters 0, 1, 2, 4, 6, 8, 9, 14 and 16 are cortical cells based on enrichment of isoflavone synthase 1 (*MtIFS1*, *Medtr4g088195*) and isoflavone synthase 3 (*MtIFS3*, *Medtr4g088160*)^97^, as well as ATP-binding cassette transporter 59 (*MtABCG59*, *Medtr3g107870*)^45^, which encodes a strigolactone transporter that is expressed in cortical cells under phosphate-depleted conditions. Cluster 14 represents cortical cells that are colonized by AM fungi, based on a range of known marker genes, including ATP-binding cassette transporter (*MtNOPE1*, *Medtr3g093270*)^98^, two isoforms of ABCB for mycorrhization and nodulation (*MtAMN2*, *Medtr4g081190* and *MtAMN3*, *Medtr8g022270*)^99^, 1-deoxy-d-xylulose 5-phosphate synthase (*MtDXS2*, *Medtr8g068265*)^46^, reduced arbuscular mycorrhiza 1 (*MtRAM1*, *Medtr7g027190*)^48^ and vapyrin (*MtVPY*, *Medtr1g089180*)^100^. We specifically focused on more mature roots, excluding meristematic or developing cells for most cell types as arbuscules do not form in these cell types. As a result, we did not observe evidence for developmental variation (trajectories) that are typically captured in single-cell studies of embryonic or meristematic tissues. Marker genes for quiescent centre and lateral root primordia, however, were used to identify cluster 12 as meristematic cells, including four homologues of plethora (*MtPLT1-4*, *Medtr2g098180*, Medtr4g065370, Medtr5g031880 and Medtr7g080460)^101^ and yucca *(MtYUC8*, Medtr7g099330). *MtYUC8*^102^ and *MtPLT* genes tend to be associated with nodule formation as well, but other genes that are upregulated by nodulation, such as nodule inception (*MtNIN*, Medtr5g099060)^103^, were either absent from our dataset or expressed at low levels and not specific to any cluster. Respiratory burst oxidase homologues (*MtRboHF*, Medtr7g060540)^104^, which is specifically expressed in root hairs, defined a small cluster adjacent to the LRP cluster as root hairs. The presence of scarecrow (*MtSCR*, Medtr7g074650)^105^ indicated that cluster 7 represents endodermal cells. Clusters 3, 5 and 11 were predicted to be vascular tissue, with several stele-specific *A. thaliana* homologues such as three homologues of transcription factor MYB domain protein (*MtMYB071*, Medtr5g014990, *MtMYB113*, Medtr2g096380, and *MtMYB112*, Medtr4g063100), as well as functionally characterized *M. truncatula* marker genes enriched in these clusters: three phosphate transporter homologues: (*MtPHO1.1-1.3*, Medt1g041695, Medtr1g075640, Medtr8g069955)^48,106^ and sugar transport protein 13 (*MtSTP13*, Medtr1g104780)^25^. Based on homologues from *Arabidopsis* marker genes, such as peroxidase 13 (*MtPrx13*, Medtr1g101830)^36^ and LOB-domain protein (*MtLBD18*, Medtr8g036085)^36^, cluster 17 represents xylem cells. *FE/*altered phloem development (*MtFe*, Medtr6g444980) and *Arabidopsis* phloem early DOF1 homologues (*PEAR1*, Medtr3g077750 and Medtr4g461080) were enriched in cluster 13, suggesting that these are phloem cells^107^. We defined cluster 15 as representing companion cells, as it is enriched for *Arabidopsis* phloem marker homologue super numeric nodules (*MtSUNN*, Medtr4g070970)^108^ and homologues of *Arabidopsis* companion cell markers *Arabidopsis* sucrose proton symporter 2 (*AtSUC2*, Medtr1g096910) and *Arabidopsis* FT interacting protein1 *(AtFTIP1*, Medtr0291s0010)^109^. Clusters 5 and 11 were enriched for *MtPHO1.1-1.3* (Medtr1g041695, Medtr1g075640 and Medtr8g069955)^106^, suggesting that these are central cylinder/pericycle cells. Marker genes for all snRNA-seq clusters can be found in Supplementary Table 13 and a dot plot showing expression of markers for each cluster can be found in Extended Data Fig. 1.

#### Differential gene expression

Differentially expressed genes (upregulated and downregulated) between the mycorrhizal and control integrated datasets for all clusters were identified using the Likelihood Ratio Test from the DESeq2 (ref. ^110^) package with an adjusted *P* value of <0.05 and a log_2_ fold change threshold of −1.0 or 1.0.

#### Module score analysis

To determine which cells represented CCCs, we generated a list of genes (Supplementary Table 3) that are known to be involved in colonization, and used these as input to assign a module score to each cell using the Seurat AddModuleScore function. Cells with a score above the threshold of 98th percentile were selected as ‘colonized’ cells and subsetted to a new object for further subclustering analysis.

#### Gene expression imputation

Using the annotated snRNA-seq integrated object as a reference and the spatial integrated object as a query, we performed a data transfer using the UMI counts from the RNA assay within the single-nuclei object as the reference data and stored the new data under a new assay called ‘imputation’. We then were able to predict gene expression within the spatial dataset using the expression values from the snRNA-seq dataset by specifying the assay to ‘imputation’ during the analysis.

#### Voxel cell type proportion prediction in spatial RNA-seq

Using the annotated snRNA-seq integrated object as a reference and the spatial integrated object as a query, we performed a label transfer using the cell type annotations as the reference data. The query dataset is then projected onto the PCA of the reference dataset and the labels are predicted.

#### Comparison with previous datasets

We compared our dataset with two prior studies (Gaude et al. 2012 and Hogekamp et al. 2013)^24,25^ that improved our understanding of gene expression changes during the mycorrhizal symbiosis between *M. truncatula* and *R. irregularis*. We wanted to include these datasets in our analysis to identify a core set of DEGs between the three different RNA-seq techniques and be able to compare and contrast the various methods in spatial transcriptomics. One major hurdle to this comparison resulted from the use of the Affymetrix Medicago GeneChip array by these two studies leading to a difference in feature identifications (IDs). We constructed an ID converter (Supplementary Table 14) to convert between the Affymetrix GeneChip, MedtrA17_4.0 and the *M. truncatula* A17 r5.0 gene IDs for a certain locus in bulk fashion using data available at refs. ^111,112^. Genes identified as common between datasets can be found in Supplementary Table 5.

#### Gene Ontology and functional enrichment analysis

For *M. truncatula*, we conducted Gene Ontology and functional enrichment analyses utilizing the PANTHER Classification system (www.pantherdb.org)^113,114^. For *R. irregularis*, we conducted Gene Ontology analysis with the Blast2Go software^115^.

### Reporting summary

Further information on research design is available in the Nature Portfolio Reporting Summary linked to this article.

### Supplementary information

Supplementary Information Legends for Supplementary Tables 1–15.

Reporting Summary

**Supplementary Table 1** Marker genes for identifying cell types for snRNA-seq dataset. **Supplementary Table 2** sNuc-RNA-seq and spatial RNA-seq data overview. **Supplementary Table 3** List of AM marker genes used as an AM-specific expression module. **Supplementary Table 4** Nuclei CCC markers. **Supplementary Table 5** Multi-dataset DEGs comparison. **Supplementary Table 6** Gene Ontology analysis results for *M. truncatula* DEGs found within the spatial datasets. **Supplementary Table 7** Gene Ontology enrichment analysis for *M. truncatula* DEGs. **Supplementary Table 8** Gene Ontology for robust *M. truncatula* genes. **Supplementary Table 9** All expressed *R. irregularis* genes. **Supplementary Table 10** Raw and normalized values for *M. truncatula* and *R. irregularis* genes expressed in spatial cluster 3. **Supplementary Table 11** *R. irrregularis* Gene Ontology terms. **Supplementary Table 12** Raw data for colonization scoring of AM fungal structures in colonization analysis. **Supplementary Table 13** Primer sets used for reverse transcription qPCR in this study. **Supplementary Table 14** Raw data for reverse transcription qPCR of AM symbiosis marker genes in colonization analysis. **Supplementary Table 15** *M. truncatula* marker genes for all single-nuclei clusters. **Supplementary Table 16** Matched IDs for the Mt4.0v1 genome, MtrunA17r5.0-ANR genome and the Medicago GeneChip Affymetrix IDs. **Supplementary Table 17** R objects for single and multiple integrated datasets.

## Data Availability

Raw feature and UMI counts for all datasets are displayed in Supplementary Table 2. Data availability on NCBI GEO (Gene Expression Omnibus) is available at https://www.ncbi.nlm.nih.gov/geo/query/acc.cgi?acc=GSE240107 (ref. ^116^). Genome assemblies for MedtrA17_4.0 (ref. ^37^) and the *Rhizophagus irregularis* Rir_HGAP_ii_V2 (DAOM 181602, DAOM 197198)^38^ were accessed at https://www.ebi.ac.uk/ena/browser/view/GCA_000219495.2 and https://www.ebi.ac.uk/ena/browser/view/GCA_002897155.1 (refs. ^117,118^), respectively.

## Code availability

All scripts used for data analysis are available on GitHub at https://github.com/kserrano109/Medicago_Rhizophagus_RNAseq^119^ and archived at Zenodo. Information on all Seurat objects used within the data analysis for all datasets can be found in Supplementary Table 15.
