# Supplementary material for: Spatial co-transcriptomics reveals discrete stages of the arbuscular mycorrhizal symbiosis
Source: Nat Plants. 2024 Apr 8;10(4):673–88. doi: 10.1038/s41477-024-01666-3 (PMC11035146; doi:10.1038/s41477-024-01666-3)
Supplement: Supplementary file 1 — Legends for Supplementary Tables 1–15. [file 41477_2024_1666_MOESM1_ESM.pdf]

nature plants

Resource https://doi.org/10.1038/s41477-024-01666-3

Spatial co-transcriptomics reveals discrete stages of the arbuscular mycorrhizal symbiosis

In the format provided by the authors and unedited

Supplementary Table Legends

Table S1. Marker genes for identifying cell types for snRNA-seq dataset. Information on the Gene ID, Gene Name, Transcript ID, Cell Type, and References (provided as DOIs) for all genes used as marker genes to identify cell type identities within the snRNA-seq cluster analysis.

Table S2. sNuc-RNA-seq and Spatial RNA-seq data overview. General information such as sequencing statistics, technology used and treatments for each individual dataset utilized in the present study. Additional information on quality control metrics used for filtering and normalization is also provided.

Table S3. List of AM marker genes used as an AM-specific expression module. Information on the Gene ID, Gene Name, Colonization Stage, and References (provided as DOIs) for all genes used as marker genes to identify colonized cells within the snRNA-seq and spatial analysis.

Table S4. Nuclei colonized cortical cell markers. Within the integrated AM-colonized snRNA-seq Seurat object, differential gene expression resulted in a list of marker genes or genes upregulated in colonized cortical cell cluster (cluster #14) relative to all other cortical cells. Information on the transcript IDs and gene description as well as expression metrics are provided.

Table S5. Multi-Dataset differentially expressed genes comparison. Filtered and matched gene IDs of transcripts from Gaude et al. 2011 and Hogekamp et al. 2013 LCM-based studies in comparison to the present study in terms of differentially-expressed genes. Different genome assembly IDs are provided as well as expression metrics for all three studies.

Table S6. Gene ontology analysis results for *M. truncatula* differentially expressed genes found within the spatial datasets. Results of gene ontology analysis search for *M. truncatula* genes found to be significantly upregulated or downregulated between the mycorrhizal and mock-inoculated capture areas within the spatial transcriptomics dataset.

Table S7. Gene Ontology Enrichment Analysis for *M. truncatula* differentially expressed genes. GO Enrichment Analysis results for all *M. truncatula* significantly up-regulated and down-regulated genes between the mycorrhizal and mock-inoculated capture areas within the spatial transcriptomics dataset.

Table S8. Gene Ontology for robust *M. truncatula* genes. GO Enrichment Analysis results for 188 robust *M. truncatula* significantly up-regulated genes between the mycorrhizal and mock-inoculated capture areas within the spatial transcriptomics dataset and also up-regulated in the Gaude et al. 2011 and Hogekamp et al. 2013 LCM-based studies.

Table S9. All expressed *R. irregularis* genes. Transcript IDs and expression metrics as well as gene ontology terms identified for all expressed *R. irregularis* genes captured by the spatial RNA-seq platform.

Table S10. Raw and normalized values for *M. truncatula* and *R. irregularis* genes expressed in spatial cluster #3. Spatial cluster #3 was identified as AM-responsive via the expression patterns of known symbiosis marker genes from both *M. truncatula* and *R. irregularis.* All transcripts found to be expressed within this cluster can be found within this table, along with raw and normalized expression values.

Table S11. *R. irrregularis* Gene Ontology terms. Results of gene ontology analysis search for *R. irregularis* captured within the spatial transcriptomics dataset.

Table S12. Raw data for colonization scoring of AM fungal structures in colonization analysis. Trouvelot colonization scoring results for all control and mycorrhizal samples analyzed. F%, frequency of infection; M%, total mycorrhization, A%, total arbuscule abundance; H%, total intraradical hyphae abundance, and V%, total vesicle abundance all provided for each sample.

Table S13. Primer sets used for qRT-PCR in this study. A list of primer sequences and purpose for all primers used in this study.

Table S14. Raw data for qRT-PCR of AM symbiosis marker genes in colonization analysis. log(2^-ΔΔCt) values for all control and mycorrhizal samples analyzed to determine successful colonization via qRT-PCR of AM symbiosis marker genes, MtPT4 and RiTUB.

Table S15. *M. truncatula* marker genes for all single nuclei clusters. Information on the Transcript IDs and expression metrics for all genes identified as marker genes for unique clusters within the snRNA-seq datasets.

Table S16. Matched IDs for the Mt4.0v1 genome, MtrunA17r5.0-ANR genome, and the Medicago GeneChip Affymetrix IDs. We constructed an ID converter (Table S14) to convert between the Affymetrix GeneChip, MedtrA17_4.0, and the *M. truncatula* A17 r5.0 gene IDs for a certain locus in bulk fashion using data available at https://medicago.toulouse.inra.fr/MtrunA17r5.0-ANR.

Table S17. R objects for single and multiple integrated datasets. R object names for single and multiple dataset integrations referenced in data analysis pipelines available at https://github.com/kserrano109/Medicago_Rhizophagus_RNAseq.
