# Supplementary material for: Spatial co-transcriptomics reveals discrete stages of the arbuscular mycorrhizal symbiosis
Source: Nat Plants. 2024 Apr 8;10(4):673–88. doi: 10.1038/s41477-024-01666-3 (PMC11035146; doi:10.1038/s41477-024-01666-3)
Supplement: Supplementary file 2 — Reporting Summary [file 41477_2024_1666_MOESM2_ESM.pdf]

nature portfolio

Corresponding author(s): Benjamin Cole, Karen Serrano

Last updated by author(s): 02/14/2023

Reporting Summary

Nature Portfolio wishes to improve the reproducibility of the work that we publish. This form provides structure for consistency and transparency in reporting. For further information on Nature Portfolio policies, see our Editorial Policies and the Editorial Policy Checklist.

Statistics

For all statistical analyses, confirm that the following items are present in the figure legend, table legend, main text, or Methods section.

n/a	Confirmed	
☐	☒	The exact sample size (*n*) for each experimental group/condition, given as a discrete number and unit of measurement
☐	☒	A statement on whether measurements were taken from distinct samples or whether the same sample was measured repeatedly
☐	☒	The statistical test(s) used AND whether they are one- or two-sided<br>*Only common tests should be described solely by name; describe more complex techniques in the Methods section.*
☒	☐	A description of all covariates tested
☐	☒	A description of any assumptions or corrections, such as tests of normality and adjustment for multiple comparisons
☐	☒	A full description of the statistical parameters including central tendency (e.g. means) or other basic estimates (e.g. regression coefficient) AND variation (e.g. standard deviation) or associated estimates of uncertainty (e.g. confidence intervals)
☐	☒	For null hypothesis testing, the test statistic (e.g. *F*, *t*, *r*) with confidence intervals, effect sizes, degrees of freedom and *P* value noted<br>*Give P values as exact values whenever suitable.*
☒	☐	For Bayesian analysis, information on the choice of priors and Markov chain Monte Carlo settings
☒	☐	For hierarchical and complex designs, identification of the appropriate level for tests and full reporting of outcomes
☒	☐	Estimates of effect sizes (e.g. Cohen's *d*, Pearson's *r*), indicating how they were calculated

Our web collection on statistics for biologists contains articles on many of the points above.

Software and code

Policy information about availability of computer code

Data collection	Zeiss ZEN Blue software was used for image capture.
Data analysis	The following software and packages were used in the analysis:<br>Cellranger 7.0<br>Spaceranger 1.3<br>R 4.2.1<br>Seurat 4.3.0<br>DESeq2<br>All software and packages can be found in the docker image: bcoli/renv_single_cell:2.1.1. Jupyter notebooks and instructions for reproducing the datasets and figures described in this manuscript can be found at https://github.com/kserrano109/Medicago_Rhizophagus_RNAseq.

For manuscripts utilizing custom algorithms or software that are central to the research but not yet described in published literature, software must be made available to editors and reviewers. We strongly encourage code deposition in a community repository (e.g. GitHub). See the Nature Portfolio guidelines for submitting code & software for further information.

Data

Policy information about availability of data

All manuscripts must include a data availability statement. This statement should provide the following information, where applicable:

- Accession codes, unique identifiers, or web links for publicly available datasets
- A description of any restrictions on data availability
- For clinical datasets or third party data, please ensure that the statement adheres to our policy

Raw and processed data files for all single-nuclei and spatial RNA-seq datasets were deposited into the Gene Expression Omnibus database under accession number GSE240107 and are available at the following URL: https://www.ncbi.nlm.nih.gov/geo/query/acc.cgi?acc=GSE240107. All data supporting the findings of this study are available within the paper and its Supplementary Information.

Research involving human participants, their data, or biological material

Policy information about studies with human participants or human data. See also policy information about sex, gender (identity/presentation), and sexual orientation and race, ethnicity and racism.

Reporting on sex and gender	n/a
Reporting on race, ethnicity, or other socially relevant groupings	n/a
Population characteristics	n/a
Recruitment	n/a
Ethics oversight	n/a

Note that full information on the approval of the study protocol must also be provided in the manuscript.

Field-specific reporting

Please select the one below that is the best fit for your research. If you are not sure, read the appropriate sections before making your selection.

☒ Life sciences ☐ Behavioural & social sciences ☐ Ecological, evolutionary & environmental sciences

For a reference copy of the document with all sections, see nature.com/documents/nr-reporting-summary-flat.pdf

Life sciences study design

All studies must disclose on these points even when the disclosure is negative.

Sample size	For the spatial RNA-seq datasets, there are 9 capture areas representing ~50 root cross sections from 3 biological and 1-4 technical replicates for mycorrhizal-treated plants and 8 capture areas representing representing ~50 root cross sections from 2 biological and 4 technical replicates for non-inoculated plants.<br>For scRNA-seq experiments, by collecting 20,000 nuclei, we expected to obtain transcriptional profiles of ~10,000 nuclei, and downselect from this the 'healthy' nuclei for downstream analysis. By repeating this across seven experiments, we obtained a dataset of 16,890 nuclei for analysis.<br>Colonization assessments were performed on five biological replicates and three technical replicates each from two harvests. 60 root fragments were assayed according to the Trouvelot scoring method from each of the 5 biological replicates for scoring of fungal presence.
Data exclusions	2 of the 7 snRNA-seq datasets were excluded due to poor apparent colonization by R. irregularis.
Replication	Three independent trials were performed each for scRNA-seq and spatial transcriptomics experiments: inoculated and non-inoculated plants grown in separate trays in the same growth chamber for each replicate. Three plants were randomly selected from 16 plants grown, their tissues pooled and processed for each experiment. Each replicate was performed non-concurrently under conditions as similar as possible. Two complementary technologies were used to probe the Medicago/AMF system, and were used to bolster findings from each platform. All replications were successful aside from the 2 snRNA-seq datasets that were not well colonized by R. irregularis as stated above.
Randomization	Roots were randomly collected and apportioned into single-nuclei populations, or randomly assorted into blocks for cryosectioning.
Blinding	Due to the nature of the experimental design and analysis pipelines, as it was our goal to draw comparisons between two treatments and not analyze each dataset independently, we felt blinding was not necessary. All colonization scoring was performed without knowledge of the sample name, thus blinding was applied here.

Reporting for specific materials, systems and methods

We require information from authors about some types of materials, experimental systems and methods used in many studies. Here, indicate whether each material, system or method listed is relevant to your study. If you are not sure if a list item applies to your research, read the appropriate section before selecting a response.

Materials & experimental systems

n/a	Involved in the study	
☒	☐	Antibodies
☒	☐	Eukaryotic cell lines
☒	☐	Palaeontology and archaeology
☒	☐	Animals and other organisms
☒	☐	Clinical data
☒	☐	Dual use research of concern
☐	☒	Plants

Methods

n/a	Involved in the study	
☒	☐	ChIP-seq
☒	☐	Flow cytometry
☒	☐	MRI-based neuroimaging

Dual use research of concern

Policy information about dual use research of concern

Hazards

Could the accidental, deliberate or reckless misuse of agents or technologies generated in the work, or the application of information presented in the manuscript, pose a threat to:

No	Yes	
☒	☐	Public health
☒	☐	National security
☒	☐	Crops and/or livestock
☒	☐	Ecosystems
☒	☐	Any other significant area

Experiments of concern

Does the work involve any of these experiments of concern:

No	Yes	
☒	☐	Demonstrate how to render a vaccine ineffective
☒	☐	Confer resistance to therapeutically useful antibiotics or antiviral agents
☒	☐	Enhance the virulence of a pathogen or render a nonpathogen virulent
☒	☐	Increase transmissibility of a pathogen
☒	☐	Alter the host range of a pathogen
☒	☐	Enable evasion of diagnostic/detection modalities
☒	☐	Enable the weaponization of a biological agent or toxin
☒	☐	Any other potentially harmful combination of experiments and agents
